# d-Amino acids and kidney diseases

**DOI:** 10.1007/s10157-020-01862-3

**Published:** 2020-02-29

**Authors:** Tomonori Kimura, Atsushi Hesaka, Yoshitaka Isaka

**Affiliations:** 1 KAGAMI Project, National Institute of Biomedical Innovation, Health and Nutrition (NIBIOHN), Osaka, Japan; 2 Reverse Translational Research Project, Center for Rare Disease Research, National Institute of Biomedical Innovation, Health and Nutrition (NIBIOHN), Osaka, Japan; 3 Department of Nephrology, Osaka University Graduate School of Medicine, Osaka, Japan

**Keywords:** d-Amino acids, Kidney disease, Early screening, Biomarker, Prognosis, d-Serine, Glomerular filtration rate

## Abstract

d-Amino acids are the recently detected enantiomers of l-amino acids. Accumulating evidence points their potential in solving the long-standing critical problems associated with the management of both chronic and acute kidney diseases. This includes estimating kidney function, early diagnosis and prognosis of chronic kidney disease, and disease monitoring. Among the d-amino acids, d-serine levels in the blood are strongly correlated with the glomerular filtration rate and are useful for estimating the function of the kidney. Urinary d-serine also reflects other conditions. The kidney proximal tubule reabsorbs serine with chiral-selectivity, with d-serine being reabsorbed much less efficiently than l-serine, and urinary excretion of d-serine is sensitive to the presence of kidney diseases. Therefore, assessing the intra-body dynamics of d-serine by measuring its level in blood and urinary excretion can be used to detect kidney diseases and assess pathophysiology. This new concept, the intra-body dynamics of d-serine, can be useful in the comprehensive management of kidney disease.

## Introduction

**Fig. 1:**
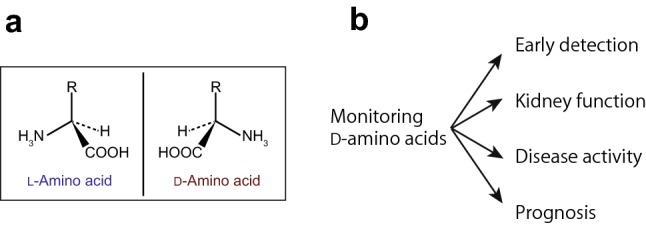
d-Amino acids and kidney disease. **a** Each amino acid, except glycine, has a chiral center and consists of two enantiomers, l- and d-amino acids. **b** Monitoring d-amino acids enables comprehensive management of kidney diseases, from early detection, assessment of kidney function, monitoring disease activity, and prediction of prognosis

Each l-amino acid has a mirror-image enantiomer (chiral body), the d-amino acid (Fig. 1a). Although its presence has long been overlooked [1, 2], recent advancement of technology is now revealing the presence of d-amino acids in nature. Remarkably, d-amino acids are emerging as biomarkers of kidney disease (Fig. 1b). Current biomarkers for kidney disease are generally accepted as insufficient [3], and d-amino acids may solve the long-lasting clinical problems of kidney diseases.

Historically, the motivation to study d-amino acids in the human body arose from the research of the kidneys. After more than 80 years of interval, the research on the role of d-amino acid in the kidneys has just re-started [4–6].

This review outlines the close relationship between d-amino acids and the kidneys, as well as recent advancements in this field.

## d-Amino acids and the kidneys

Amino acids are the smallest components of proteins. When synthesized chemically, equal amounts of l- and d-amino acid are produced. However, d-amino acids were not previously detected in nature, and thus, it has long been believed that only l-amino acids existed. Recently, d-amino acids have been found in nature and even in the human body. Moreover, d-amino acids were shown to have physiological activities despite their trace levels [7, 8], and thus, studies of d-amino acids are currently underway.

It was the kidney that the presence of d-amino acids was first indicated in mammals. In 1935, Dr. Hans Krebs, who discovered the Krebs’ cycle (also known as citrate cycle or tricarboxylic acid cycle), found that the kidneys contain an enzyme that degrades d-amino acids [1]. On purifying this enzyme, later termed as d-amino acids oxidase (DAO), Dr. Krebs predicted the presence of d-amino acid in the kidneys. Unfortunately, it was already believed that the human body contains only l-form amino acids. Therefore, DAO was considered as functionless. Some nephrologists disagreed with this misconcept and attempted to demonstrate the presence of d-amino acids in body (personal communication); however, d-amino acids had not been detected in humans. Some earlier studies reported the presence of d-amino acids in patients with kidney diseases [2, 9–11], but methods for precisely detecting trace amounts of d-amino acids in human precisely were not available.

## Measurements of d-amino acids

A main limitation of the studies of d-amino acids is their measurement. Only trace amounts of d-amino acids are present as compared to the abundant amounts of l-amino acids in nature and in the human body. In addition to l-amino acids, a wide variety of intrinsic compounds is present in human samples, which can easily interfere with analyses due to, again, trace nature of d-amino acids. These features of d-amino acids often prevent their precise measurements.

Precise measurements are key in biomarker identification. Biomarker mining using precision measurement reduces false-positive and false-negative results, greatly reducing the efforts of researchers. Biomarker analysis can affect patient outcome, i.e., uncertainty or wider deviations in the measurement lead to ambiguous diagnosis, making treatment difficult and causing anxiety in patients. Even if potential targets are identified, the development of precision methods for detecting specific targets is eventually necessary.

Recently, precise measurement of d-amino acids has become possible using two-dimensional high-performance liquid chromatography (2D-HPLC) [12, 13]. The 2D-HPLC system is comprised of two tandemly aligned HPLC steps; the first dimension separates each amino acid using a reversed-phase column, and the second dimension separates each enantiomer using a chiral column. 2D-HPLC can precisely detect all amino acids with enantiomer selectivity over a range from 1 fmol to 100 pmol.

## Clinical problems involving d-amino acids

Based on the close relationship between d-amino acids and the kidney, the key problems in kidney disease research are being investigated based on d-amino acids.

### Prediction of prognosis of chronic kidney disease (CKD)

CKD, often defined by a chronic reduction in kidney function, glomerular filtration rate (GFR) [14], is a global concern with more than 10 million patients in Japan and 850 million worldwide [15, 16]. The management of CKD is critical for suppressing the onset of cardiovascular diseases and progression to end-stage kidney disease (ESKD); however, satisfactory methods are lacking for early detection and prediction of prognosis.

**Fig. 2:**
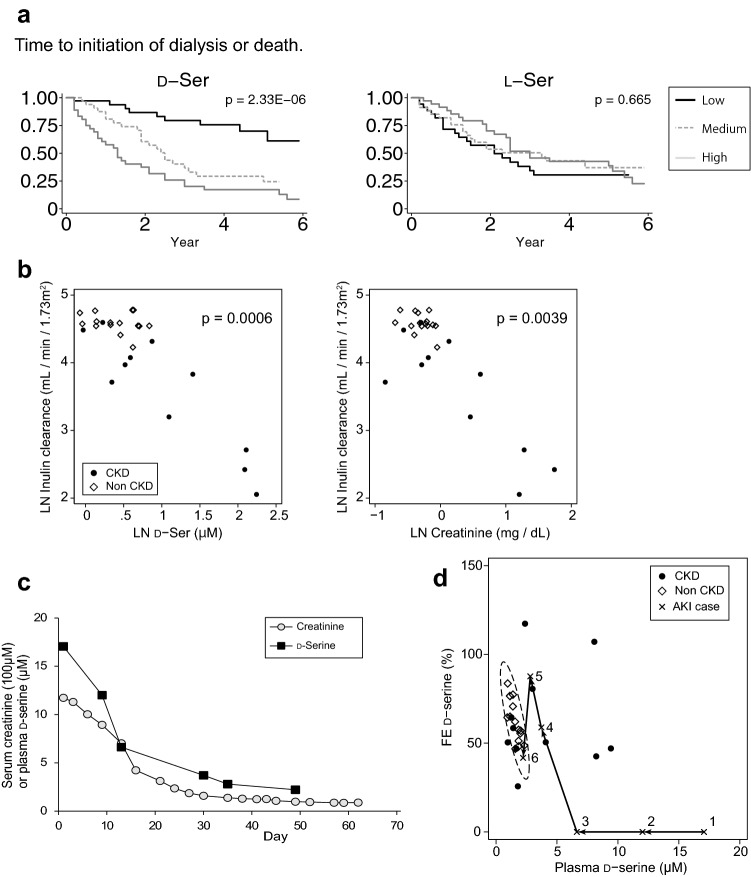
Monitoring intra-body dynamics of d-serine for assessment of the kidney diseases activity. **a** Higher blood levels of d-serine are associated with worse prognosis of patients with CKD. Note that this trend is chiral-selective and is not observed in l-serine. **b** Blood levels of d-serine are well correlated with inulin clearance, the golden standard for glomerular filtration rate, a key feature in predicting GFR. This correlation is compatible with that of creatinine. *LN* log-natural transformed. **c** In the recovery phase of a patient with acutely worsened kidney, blood levels of d-serine dynamically responded to treatment. This trend paralleled the changes in blood creatinine. **d** Plotting of intra-body dynamics of d-serine indexed by plasma levels and fractional excretions (FE). The profile of non-CKD conditions has been represented to be restricted within a certain range (dotted eclipse, 95% CI), whereas that of CKD condition has been represented to be broad and mostly outside the eclipse. The profile shifts from 1 to 6 in the recovery course of acutely worsened kidney. Figures are adopted from references [3–5] with modifications

The first CKD problem tackled by d-amino acids is the prediction of the prognosis (Fig. 2a) [4]. In a longitudinal cohort of 108 patients with CKD, 2D-HPLC-based metabolomics analysis was performed to determine its predictive values for CKD prognosis. Sixteen of 21 measured d-amino acids were detected in the blood of patients with CKD. Among them, d-serine, d-alanine, d-proline, and d-asparagine showed the highest detection rates.

Survival analysis revealed a relationship between d-amino acids and the prognosis of CKD; patients with higher blood levels of d-amino acids are likely to have progressive CKD and start dialysis early. Among the d-amino acids, d-serine and d-asparagine were strongly associated with a worse prognosis. The risk of progression to ESKD was two-to-fourfold higher in patients with higher levels of these d-amino acids.

Blood d-amino acids show potential for predicting the prognosis of patients with CKD. Measuring d-amino acids may help to distinguish patients with CKD at high risk and prioritize those in need of more intensive care. This study provides new research opportunities in the fields of nephrology and use of d-amino acids as CKD biomarkers.

### Estimation of GFR and early screening of CKD

After successfully predicting CKD prognosis using d-amino acids, next problem of CKD, early screening, was investigated (Fig. 2b) [5]. Precise evaluation of kidney function based on GFR is key for early screening, but has not been achieved. Inulin clearance, the gold standard of GFR, is less frequently measured in clinics due to the complexity of the measurement. The estimated GFR (eGFR) based on the measurements of creatinine and cystatin C is more convenient and widely used, but shows limited accuracy [17] [18]. Previously, a close correlation between the blood level of d-serine and eGFR has been reported [4].

A study examined the potential of using d-serine as a kidney GFR marker [5]. This study was performed to measure inulin clearance and blood d-serine simultaneously in CKD patients and non-CKD subjects [5]. The results of this study showed that the blood levels of d-serine were well correlated with inulin clearance. This correlation was equivalent to those of the conventional kidney disease markers, creatinine, and cystatin C. d-Serine was identified as a useful marker for estimating the GFR and, thus, for the early diagnosis of CKD.

### Intra-body dynamics of d-serine: another early screening method for CKD

Mechanism of d-serine management by the kidneys and the dynamics of d-serine in the body need to be understood (Fig. 2c) [5]. To understand this, fractional excretion (FE) of d-serine was examined [5]. The FE is the ratio of urinary excretion over glomerular filtration of a certain substrate. In case of l-amino acids, for example, the FE is about 1%, suggesting that 99% of l-amino acids filtrated in the glomerular are reabsorbed [19]. Most l-amino acids are reabsorbed, as they are important nutrients and need to be recovered from urinary excretion.

Surprisingly, the mode of urinary excretion of d-serine greatly differs from that of its enantiomer. The FE of d-serine was 62% in median in non-CKD subjects, which was much higher than that of l-serine (1.3%). The kidney clearly handles amino acids with chirality.

Interestingly, the FE of d-serine shows a similar in median but a wider range in patients with CKD as compared with that of non-CKD. The FE of d-serine is restricted within a certain range in non-CKD subjects. Principal component analysis revealed that the FE of d-serine conveyed unique information unrelated with the d-serine level in blood. Specifically, blood d-serine clustered in the group formed by the conventional kidney GFR markers creatinine and cystatin C, further reflecting its character as a kidney marker. The FE of l-amino acids formed another cluster, reflecting the similar mode of urinary l-amino acid dynamics regulated by the kidney. However, the FE of d-serine showed characteristics distinct from both kidney GFR markers and l-amino acid dynamics.

The FE of d-serine turned out to show high sensitivity in the presence of CKD, suggesting that the FE is useful for screening of kidney diseases. This was confirmed by analyzing the intra-body dynamics of d-serine, indexed by the blood and FE of d-serine (Fig. 2c). Intra-body dynamics of d-serine showed similar profiles in non-CKD subjects but different and diverse profiles in patients with CKD. Some patients with CKD showed a plasma-normal but FE-high profile of d-serine. The kidney function of these patients was found to be within the normal range, as reflected by the normal range of the d-serine plasma level. In these patients, eGFR alone cannot detect CKD. However, the abnormal FE of d-serine facilitated detection of CKD in these otherwise apparently healthy subjects. Therefore, the FE of d-serine provides additional information for detecting CKD even in cases when eGFR range appears to be normal. Detecting CKD before eGFR is decreased is an important problem in clinical detection of CKD, which may be overcome by measuring the FE of d-serine.

Another important aspect which may be provided by intra-body dynamics of d-serine is the sensitivity of CKD diagnosis. We now know that CKD is prevailing throughout the world [15, 16]. Estimation of GFR has opened the field of early screen [14]; however, the diagnostic accuracy has been left unsolved. In the medical diagnosis, specificity of a biomarker is used for early screen, whereas its sensitivity is used for accurate diagnosis. To gain sensitivity in CKD diagnosis is the next step to overcome. The sensitivity of intra-body dynamics for the diagnosis of CKD was reported to be high (72.7%) [5]. High sensitivity of intra-body dynamics of d-serine may support the correct diagnosis of CKD.

In summary, d-serine is useful for the early detection of CKD from two perspectives. GFR can be estimated by determining blood levels of d-serine. Additionally, assessment of the intra-body dynamics of d-serine, by combining evaluation of the blood levels and FE of d-serine, is useful for detecting CKD before GFR declines.

### Assessment of disease activity

The intra-body dynamics of d-serine also reflect the activity of kidney disease [6]. In a patient with acutely worsened kidney function due to systemic lupus erythematosus, the blood levels of d-serine were extremely high before treatment (17.06 μM), because kidney filtration function was completely impaired. Upon treatment, the blood levels of d-serine dynamically responded and became normalized, which paralleled the levels of blood creatinine (Fig. 2d). Currently, blood creatinine is used to assess the disease activity of acute kidney injury [20], but its measurement is often limited by the time lag of the response [21]. Thus, blood d-serine may be useful for the assessment of disease activity.

The FE of d-serine, another factor in the intra-body dynamics of d-serine, also provided key information (Fig. 2c). Before treatment of the patient, the FE of d-serine could not be detected because of its extremely low level in contrast with 60% in the median value found in the non-CKD subjects. As kidney function was recovered, the FE of d-serine transiently over-surged the normal level, followed by normalization. This response suggests that the kidney adaptively increased urinary excretion of d-serine to normalize the level in blood. Therefore, the FE of d-serine responded to disease activity during the clinical course. Accordingly, monitoring the intra-body dynamics of d-serine is useful for assessing disease activity and may improve prognosis and determination of treatment efficacy.

## Key determinant of intra-body dynamics

**Fig. 3:**
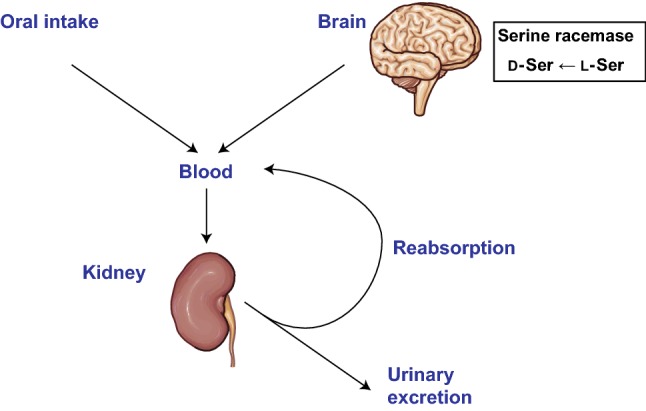
Regulation of intra-body dynamics of d-amino acids by the kidneys. d-Amino acids, either orally consumed or produced by serine racemase in case of d-serine, are delivered to the kidneys. Filtrated d-amino acids by the kidneys are either excreted via urine or reabsorbed to re-enter blood circulation. Images are modified from Servier Medical Art, licensed under a Creative Commons Attribution 3.0 Generic License. https://smart.servier.com/

Monitoring the intra-body dynamics of d-serine provides comprehensive information for managing kidney diseases (Fig. 1b). Then, what are the determinants of d-serine dynamics (Fig. 3)?

The major determinant is blood flow to the kidneys. Once GFR is reduced, the kidneys cannot filter d-serine and it is, therefore, not excreted via urine. As a result, blood level of d-serine increases. This likely explains why blood levels of d-serine are correlated with GFR [5].

Another determinant of d-serine dynamics is tubular reabsorption. As aforementioned, a large fraction of d-serine is excreted into urine; the FE is 60% for d-serine vs 1% for l-serine [5]. Reabsorption of d-serine occurs in the proximal part of the tubules, and therefore, the presence of transporters for d-serine in this part was suggested [22, 23]. These transporters, potentially either d-amino acid selective or non-selective, are assumed to be less efficient based on higher excretion and lower uptake of d-serine [5]. Recovery of FE of d-serine after AKI strongly suggests that tubular injury affects the reabsorption process of d-serine [6].

Interestingly, reabsorption of d-serine changes dynamically and is sensitive to the presence of underlying diseases. In the early stage of CKD when kidney function and blood levels of d-serine are normal, the kidneys adaptively respond by increasing the excretion of d-serine to potentially maintain the level of d-serine in blood. In later stages with reduced blood flow to the kidneys and reduced GFR, the kidney tubules cannot manage d-serine levels because of decreased influx. Thus, the blood level of d-serine increases when the adaptive response of the kidneys is insufficient.

## Origin of d-serine

To fully utilize the role of d-serine as a biomarker, it is important to understand its source and general role in the body (Fig. 3). One key source of d-serine is serine racemase (SR) in the neurons of the forebrain [24, 25], which catalyzes the stereochemical inversion of l-serine to d-serine [26]. Another enzymatic regulator is DAO [1]. DAO oxidizes d-serine and generates 2-hydroxy-pyruvate along with hydrogen peroxide and ammonia [27, 28].

SR and DAO show different tissue distributions, which may regulate the dynamics of d-serine. SR is predominantly expressed in the neurons of the forebrain [24, 25]. In agreement with the localization of the enzyme, d-serine is most abundant in cerebrum [13, 29]. In the brain, d-serine serves as a neurotransmitter of *N*-methyl-d-aspartate (NMDA)-type glutamate receptor (NMDAR) which maintains synaptic plasticity [30].

While SR is predominant in the forebrain, DAO is expressed more broadly [27, 28]. DAO is most abundant in the kidney. DAO is also present in liver except mouse liver. In the rodent brain, DAO activity is restricted to astrocytes in the hindbrain and spinal cord. DAO activity is also detected in the neutrophils, retina, and the small intestine in mouse. The complemental distribution of SR and DAO in the brain may determine the heterogenic distribution of d-serine [31].

Excessive oral intake of d-serine directly increases its levels in blood [32]. Some foods contain d-serine synthesized during the fermentation processes [33]. Intestinal microbiota can affect the intra-body d-amino acid levels by similar processes. Some d-amino acids such as d-alanine, d-asparagine, d-glutamate, and d-proline were detected in the feces of specific pathogen-free mice, but not in those of germ-free mice [34], suggesting that the microbiota in the intestine affects the d-amino acid profile. Intestinal microbiota may also affect intra-body d-serine levels [34], although d-serine-producing microbiota are yet to be identified.

## Physiological function of d-serine in kidney

As mentioned, researchers have been interested in the presence of DAO in the kidney; the physiological significance of d-serine in kidney has not been determined. The blood d-serine level is regulated within a narrow range by the kidney as seen in human studies [4–6], possibly to avoid unnecessary physiological responses to d-serine.

Previous studies showed the toxic effects of d-serine. Excessive amounts of d-serine administered in the body either intravenously or orally caused severe acute kidney injury in rodents [35, 36]. d-Serine administration caused extensive necrosis of the proximal tubules within a few hours. The mechanisms of these effects are unclear. Degradation of d-serine by DAO may play a key role in kidney toxicity [37], typically through the production of hydrogen peroxide and reactive oxygen species [38]. In contrast, administration of d-alanine, which can also produce hydrogen peroxide upon oxidation by DAO, does not induce kidney injury in rodents, obscuring the causal relationship of DAO with kidney injury [37, 39]. Using kidney tubular cell lines (HK-2) and primary cultures of kidney tubular cells, one study suggested the toxic mechanism of higher doses of d-serine as the induction of cell cycle arrest, pro-inflammatory response, and apoptosis via effector molecules of ER stress [40]. Higher doses of d-serine have been used in some clinical studies in schizophrenia and cerebellar ataxia [41, 42], as a potential activator of NMDAR. In these studies, potential side-effects such as proteinuria and abnormal kidney values were sporadically reported, although the causal relationship remains unclear [43, 44].

Another study suggested the potential protective effects of d-serine [45]. Although protective effects have not been demonstrated using the conventional kidney marker, creatinine, d-serine was reported to suppress kidney tubular damage according to the histological assessment of a rodent acute kidney injury model (ischemia/reperfusion model). The mechanisms by which d-serine exerts toxicity or protective effects are largely unsolved.

## Concluding remarks

The close relationship between d-amino acids and the kidneys is now emerging. Currently, d-serine is most deeply studied d-amino acids due to its strong potentials and relative abundance. Therefore, evidence regarding d-serine in kidney diseases is accumulating. On the other hand, such d-amino acids as d-asparagine, d-alanine, and d-proline also have prognostic impacts on the prognosis of CKD. Studies of d-amino acids in kidney diseases have just begun, and we are foreseeing that more clinical evidence of d-amino acids will accumulate with the further advancement of the technology.

d-Serine is now regarded as a potential biomarker in a wide range of kidney diseases. d-Serine reflects the activity of kidney diseases, and can be used for the detection of diseases or the prediction of prognosis, and estimation of GFR. Monitoring the intra-body dynamics of d-serine provides a new parameter for kidney diseases in clinics. Some key questions remain unsolved. For example, can combination of d-serine and current kidney markers, creatinine and cystatin C, improve the precise estimation of GFR? Is monitoring of intra-body dynamics of d-serine useful to improve the diagnosis of CKD? What kind of information do intra-body dynamics of d-serine reflect in association with other parameters of kidney diseases such as proteinuria? Is additional monitoring of other d-amino acids also useful in kidney and other diseases? These are important questions that will be answered in the future study.

Before the clinical application, it is necessary to develop a high-throughput system for measuring d-serine levels in the body. This system requires precision measurements, as the results will affect treatment decisions. With the development of research, d-serine is now solving two major problems that CKD processes, early screening and prediction of prognosis, and may lead to solve the last question, the therapy of CKD.
